# A Comparison of *rpoB* and 16S rRNA as Markers in Pyrosequencing Studies of Bacterial Diversity

**DOI:** 10.1371/journal.pone.0030600

**Published:** 2012-02-15

**Authors:** Michiel Vos, Christopher Quince, Agata S. Pijl, Mattias de Hollander, George A. Kowalchuk

**Affiliations:** 1 Department of Microbial Ecology, Netherlands Institute of Ecology (NIOO-KNAW), Wageningen, The Netherlands; 2 Department of Civil Engineering, University of Glasgow, Glasgow, United Kingdom; 3 Institute of Ecological Science, Free University of Amsterdam, Amsterdam, The Netherlands; Argonne National Laboratory, United States of America

## Abstract

**Background:**

The 16S rRNA gene is the gold standard in molecular surveys of bacterial and archaeal diversity, but it has the disadvantages that it is often multiple-copy, has little resolution below the species level and cannot be readily interpreted in an evolutionary framework. We compared the 16S rRNA marker with the single-copy, protein-coding *rpoB* marker by amplifying and sequencing both from a single soil sample. Because the higher genetic resolution of the *rpoB* gene prohibits its use as a universal marker, we employed consensus-degenerate primers targeting the Proteobacteria.

**Methodology/Principal Findings:**

Pyrosequencing can be problematic because of the poor resolution of homopolymer runs. As these erroneous runs disrupt the reading frame of protein-coding sequences, removal of sequences containing nonsense mutations was found to be a valuable filter in addition to flowgram-based denoising. Although both markers gave similar estimates of total diversity, the *rpoB* marker revealed more species, requiring an order of magnitude fewer reads to obtain 90% of the true diversity. The application of population genetic methods was demonstrated on a particularly abundant sequence cluster.

**Conclusions/Significance:**

The *rpoB* marker can be a complement to the 16S rRNA marker for high throughput microbial diversity studies focusing on specific taxonomic groups. Additional error filtering is possible and tests for recombination or selection can be employed.

## Introduction

The small subunit ribosomal RNA gene is the recognized gold standard for estimating the phylogenetic diversity in microbial communities (e.g. [1], [2], [3]). This marker gene is universally present and has the advantage of containing both highly conserved fragments, facilitating the design of PCR primers targeting all members of a community, and more variable regions that allow for the discrimination of different microbial taxa. Furthermore, the identity of 16S rRNA gene sequences collected from the environment can be related to the taxonomic identity of sequences obtained from cultivated, characterized strains. With the introduction of high throughput (pyro)sequencing methods in studies of microbial diversity, large datasets are rapidly accruing that allow patterns of sequence recovery to be examined in depth across multiple habitats and samples [4]. However, the 16S rRNA gene is not without potential drawbacks, and the use of alternative markers has been proposed, including the beta subunit of DNA polymerase, *rpoB* [5], [6], [7], [8], [9], [10].

The use of the *rpoB* gene offers various potential advantages over standard 16S rRNA gene-based approaches. First, since most bacterial genomes contain multiple copies of the 16S rRNA gene, and copy number varies per species, extrapolation of relative abundances from gene recovery frequencies is seriously impaired. This is further complicated by the fact that sequence variation between the different 16S rRNA gene copies present exists in some genomes [11], [12]). *rpoB* typically occurs in a single copy [9]. Second, the high level of conservation across 16S rRNA genes can obscure most intraspecific, and sometimes interspecific (e.g. [13]) variation. In contrast, the higher resolution *rpoB* marker is capable of revealing molecular variation down to the population level [6]. Third, genetic divergence of *rpoB* correlates better with overall genomic divergence and provides better bootstrap support for phylogenetic reconstruction [6]. Fourth, given the fact that *rpoB* is a protein-encoding gene, the data generated from this marker is more readily interpreted in an evolutionary framework. Fifth, (pyro)sequencing error is an important confounding factor in studies of microbial diversity using 16S rRNA gene sequences [14], [15], [16]. Given that *rpoB* is single-copy, essential protein-encoding gene, sequence errors can be readily identified and removed if they introduce disruptions in reading frame.

Here, we test the performance of the *rpoB* gene as a marker for pyrosequencing-based assessments of bacterial diversity in soil, and compare results using this marker to those obtained by the conventional 16S rRNA gene marker. Due to the lower degree of conservation within the *rpoB* gene and our desire to provide deep sampling, we chose to restrict the current study to a single, important bacterial phylum, namely the Proteobacteria. To this effect, consensus-degenerate PCR primers were developed for the amplification of proteobacterial *rpoB* gene fragments for direct 454-based pyrosequencing. After the resulting sequences were subjected to denoising [15], [17], it was examined how diversity estimation was improved by additional reading-frame correction. We examined the performance of both markers with respect to quantifying diversity of the proteobacterial fraction of the community. Lastly, we explored the potential of using *rpoB* gene sequences to yield insights into population-level processes.

## Materials and Methods

### Sample Collection and DNA Extraction

A single soil sample was collected from the experimental grassland field ‘De Ossekampen’ in Wageningen, The Netherlands (51°58′14″N; 5°38′19″E) and stored at −20°C for 6 days before DNA extraction. After manually removing plant material, DNA was extracted from twelve 0.25 g subsamples using the Mo-Bio PowerSoil DNA Isolation kit (Mo-Bio Laboratories) after which the DNA was pooled. DNA extraction was performed according to the manufacturer's instructions, with the only modification being that the soil suspension was heated to 65°C for 5 minutes followed by 10 minutes horizontal shaking (Retsch mixer mill, 30 Hz).

### PCR Amplification and Pyrosequencing

The V4 region of the 16S rRNA gene was amplified using forward primer 515f (5-GCCTTGCCAGCCCGCTCAGGTGTGCCAGCMGCCGCGGTAA-3) containing the 454 Life Sciences primer B, the broadly conserved bacterial primer 515F, and a 2-base linker sequence ‘TC’, and using reverse primer 806r (5-GCCTCCCTCGCGCCATCAGGGGGACTACVSGGGTATCTAAT-3) containing the 454 Life Sciences primer A and the bacterial primer 806R [18].

A fragment of the *rpoB* gene (region in-between primers binding sites corresponding to *E. coli* K-12 nucleotide positions 1671-2049) was amplified using forward primer 1f (5-GCCTTGCCAGCCCGCTCAGTCCGTGCACCCCACCcaytayggnmg-3) containing 454 Life Sciences primer B, a 2-base linker sequence ‘TC’ and consensus-degenerate primer CGTGCACCCCACCcaytayggnmg, and using reverse primer 1r (5- GCCTCCCTCGCGCCATCAGCCCACGGCCTGCckytgcatrtt-3) containing 454 Life Sciences primer A, a 2-base linker sequence ‘CC’ and consensus-degenerate primer CACGGCCTGCckytgcatrtt. The consensus degenerate primers were designed using the program iCODEHOP [19] on the basis of an alignment of 39 diverse Proteobacterial species (Table S1). This approach combines a 3′ fully degenerate primer spanning three to four codons with a longer 5′ consensus clamp containing most probable codons for that region. The pyrosequencing primer was actually not used during 454 sequencing because another primer was ligated by the sequencing company (rendering our primers longer than necessary for PCR).

PCRs contained 0.5 µl (30 pmol/µl) of each forward and reverse primer, 1 µl template DNA, 2.5 µl 10× PCR Buffer, 2.5 µl dNTP's (2 mM), 17.8 µl ddH_2_O water and 0.2 µl of FastStart Expand TAQ DNA polymerase (5 units/µl) (Roche). Samples were initially denatured at 95°C for 5 min, then amplified using 20 cycles for the 16S RNA gene and 30 cycles for the *rpoB* gene (30 s at 95°C, 45 s at 55°C and 90 s at 72°C plus a final extension of 15 mins. at 72°C to ensure complete amplification of the target region). 96 and 192 PCR amplifications were performed for the 16S rRNA- and *rpoB* genes, respectively, after which samples were pooled. Using many separate PCRs instead of one reaction, allowed us to minimize the number of PCR cycles and therefore potential PCR-induced bias and error.

Pooled samples were precipitated by adding 2 µl of 3 M NaOAc (pH 5.2), 1 µl of 100 mM Na_2_EDTA (pH 8.0) and 1 µl 20 µg/µl glycogen to 20 µl of PCR reaction and 2 volumes (96×23 µl and 192×23 µl respectively) of ice-cold 96% ethanol, followed by vortexing. Precipitated DNA was centrifuged at 3838×*g* for 10 min at 4°C, and the resulting pellet was washed in ice-cold 70% ethanol followed by another round of centrifugation. After removal of the supernatant, the pellet was dried in a speedvac (Thermoscientific) and resuspended in 125 µl of PCR grade ddH_2_O.

Because a second, low quantity amplicon was present in the PCRs targeting the *rpoB* gene, these samples were run on a 2% agarose gel and the desired band excised and purified using the Qiaquick Gel Extraction kit (Qiagen). The 16S rRNA gene PCR products were purified using the Qiaquick PCR Purification kit (Qiagen). Both samples were subjected to pyrosequencing on a 454 Life Sciences Genome Sequencer FLX (Roche) machine at ¼ plate capacity each by Macrogen Inc. (Seoul, Korea). All sequences were submitted in the MG-RAST database: http://metagenomics.anl.gov/linkin.cgi?metagenome=4476877.3 (*rpoB*) and http://metagenomics.anl.gov/linkin.cgi?metagenome=4476876.3 (16S rRNA).

### PCR and Sequencing Error Correction

Raw 16S rRNA and *rpoB* 454 reads were filtered for reads associated with errors by removing all sequences that did not have a perfect match to the degenerate primer. Reads where the first noisy flow (0.5–0.7) occurred before position 360 out of 800 were also removed. The reads were then truncated to a length of 720 flows. The filtered reads were subsequently denoised using the AmpliconNoise-Perseus pipeline [17]: 454 errors were removed by the flowgram clustering program PyroNoise [15], forward primers were removed from forward reads, the reverse primer was removed from the reverse reads and PCR point mutations were removed by the sequence clustering program SeqNoise. The chimera classifier Perseus was then used to identify the probability of each read being chimeric through rigorous search against possible parents. Any read with a 50% or greater probability of being chimeric was removed. Finally, reverse primers were removed from forward reads and vice versa.

In addition to filtering and denoising, a further level of error correction was employed for the *rpoB* marker by translating nucleotide sequences into amino acids and removing any sequences possessing nonsense mutations. As a last check, any sequences containing obviously aberrant stretches of amino acids were manually removed after visual inspection of colour-coded alignments in MEGA 4 [20] (http://www.megasoftware.net/mega4/mega.html). Lastly, the reading frame correction method was employed on the undenoised dataset to allow comparison of the two methods.

### OTU Construction and Taxonomy

OTUs (Operational Taxonomic Units) were constructed from distances calculated following exact pairwise alignment by the Needleman-Wunsch algorithm [21]. These pairwise distances were used in a complete linkage hierarchical clustering to generate OTUs at different nucleotide sequence difference cut-offs. OTUs were constructed for both the 16S rRNA and *rpoB* forward and reverse cleaned sequences. In addition, OTUs were calculated for the two genes using both forward and reverse sequences; the pairwise alignments and resulting distances ignore terminal gaps, allowing composite OTUs to be produced. The most frequent sequence in an OTU was then identified for every cluster. The 16S rRNA and *rpoB* sequence reads were classified by employing the same database (1477 prokaryote genomes; NCBI Complete Microbial Genomes, February 2011), BLAST type (nucleotide) and classification algorithm (LCA; using the default parameters in MEGAN [22]).

### Estimation of Species Diversity

Both markers can be compared at the level of total diversity quantified by each, but, since the *rpoB* marker was specifically designed to target Proteobacteria, the comparison is more meaningfully made when restricted to this phylum. Following OTU construction at 2.3% sequence difference for *rpoB* and 1% for the 16S rRNA gene (see Results for a justification of OTU cut-off values), we compared OTU diversity for reads classified as Proteobacteria. Rarefaction curves were calculated to show the effect of sample size on observed diversity. To estimate the total diversity in the community, a Bayesian method was used to fit taxa abundance distributions to the observed OTU frequencies [23]. As predictions have previously been found to be sensitive to the choice of distribution [23], both the log-normal and Sichel distributions were used.

### Population Genetic Analyses

Partial *rpoB* gene sequences classified by MEGAN as belonging to the same species were selected for population-level analyses. These DNA sequences were then aligned at the amino acid sequence level using the Clustal algorithm in MEGA 4 [20]. A Minimum Spanning Tree based on nucleotide differences was subsequently constructed using HapStar (http://www.fo.am/hapstar) [24]. A NeighborNet network analysis was constructed using SplitsTree4 (http://www.splitstree.org) [25]. The PHI test of homologous recombination [26] was also conducted using SplitsTree4.

## Results

### Sequence Error Correction

**Figure 1:**
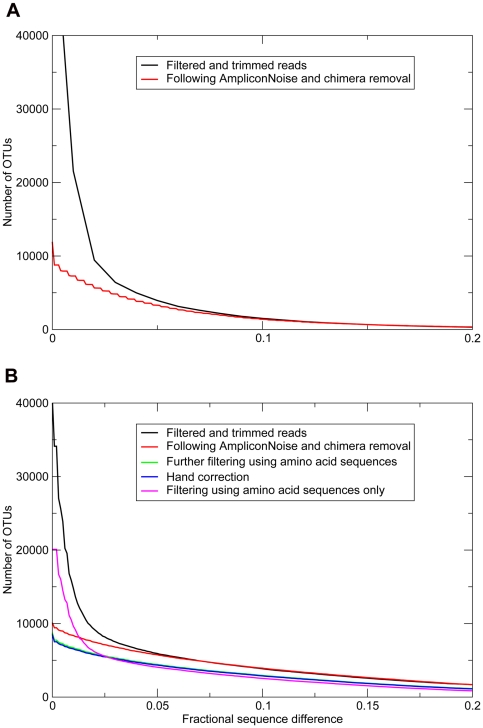
Number of OTUs as a function of fractional sequence difference (OTU cut-off) for the 16S rRNA marker gene (A) and the *rpoB* marker gene (B). OTU number is plotted for filtered and trimmed sequences (undenoised), denoised sequences and denoised- and reading frame corrected sequences (automated and manual correction). The latter treatments were only applicable to *rpoB*.

As has been demonstrated before [17], noise removal by AmpliconNoise and Perseus significantly reduced observed OTU richness. This is clearly displayed in Fig. 1, where the number of unique Operational Taxonomic Units (OTUs) for denoised and undenoised 16S rRNA and *rpoB* marker genes are plotted as a function of OTU cut-off value. Subsequent to denoising, *rpoB* nucleotide sequences were translated into amino acid sequences and any sequences containing nonsense mutations (i.e. containing stop codons or frame shifts) were removed, followed by back translation. After this automated step, 382 sequences out of 31,684 were manually removed as they were visually very dissimilar (Fig. 1B). At the level of unique sequences (0% OTU cut-off), this reading frame correction lowered observed denoised diversity with an additional 1.2%. Although this effect is small compared to the effect of denoising (which removed 27.6% of unique OTUs), the resulting data were largely freed of artefactual sequences that could negatively affect downstream population genetic analyses. When reading frame correction was applied to undenoised sequences, an excess of OTUs was observed above the 2.3% species cut-off compared to the combined use of denoising and reading-frame correction, but OTU richness was nearly identical for both approaches below this cut-off (Fig. 1B).

### Observed and Estimated Diversity

Our soil sample displayed a taxonomic composition characteristic for temperate soils [27] with abundant Proteobacteria, Acidobacteria and Actinobacteria as determined from the 16S rRNA gene sequences (Fig. S1). In order to compare the overlap in sequence target of the *rpoB* and 16S rRNA primers, we compared taxonomies using the same database and classifier (Fig. S1). It was obvious that the *rpoB* primers did not exclusively target Proteobacteria, with for instance large numbers of the unrelated Actinobacteria also amplified. The distributions among the proteobacterial classes also exhibited differences, which is to be expected as primer sets will inevitably have distinct biases (Fig. S1).

**Figure 2:**
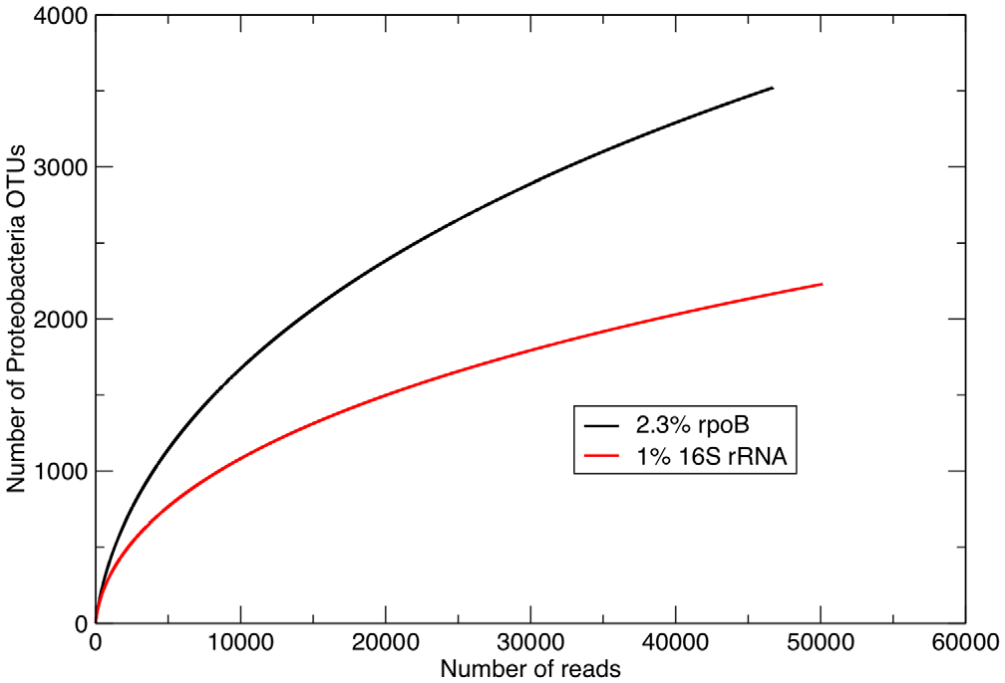
Rarefaction curves showing mean expected OTU number for Proteobacteria as a function of sample size. The 1% and 2.3% cut-offs for 16S rRNA and *rpoB* are chosen to reflect species definitions (see text).

Proteobacterial diversity was quantified at the OTU cut-off level that corresponded to an average DNA-DNA Hybridization (DDH) value of 70% used to delineate bacterial species [6], [7]. This value is 1% for the 16S rRNA gene [28] (although wider cut-offs are commonly employed for this gene as well). A value of 2.3% *rpoB* divergence has been shown to correspond best to an average 70% DDH value [7] (this also held true for the fragment of the *rpoB* gene utilized here, data not shown). Figure 2 shows an accumulation curve plotting the number of proteobacterial OTUs as a function of sampled reads, using the 16S rRNA and *rpoB* species definition OTU cut-offs. It is immediately apparent that the curves are very different, with the 16S rRNA marker indicating a distribution skewed towards more frequently occurring species (the slower accumulation of OTU richness being caused by repeated sampling of identical OTUs). A considerably higher number of proteobacterial species were found using the *rpoB* marker (Table 1).

**Table 1:** Estimation of species richness for sequences classified as Proteobacteria.

marker (OTU cut-off)	reads	observed n species	estimated n specieslog-normal distribution*	estimated n species Sichel distribution*	estimated n species Chao estimator
*rpoB* (2.3%)	46,704	3521	15,501 : 19,897 : 25,829	7299 : 8166 : 9401	5646
16S rRNA (1%)	50,145	2231	12,384 : 18,900 : 42,824	5036 : 5978 : 7699	3490

* = The parametric total diversity estimates are given as lower 95% confidence interval : median : upper 95% confidence interval.

The total proteobacterial species richness for both markers was estimated using fitting of the Sichel and lognormal distributions, as well as using the more conservative, non-parametric Chao estimator (Table 1). Both the log-normal and Sichel distributions yielded a greater total richness for *rpoB* sequences as compared to 16S rRNA gene sequences, but since confidence intervals of the two estimators overlap, this difference was not significant. The log-normal distribution was a marginally, but not significantly, better fit than the Sichel distribution for the *rpoB* data, but the converse was true for the 16S rRNA gene.

The proteobacterial OTU frequency-abundance distributions for the *rpoB* and 16S rRNA gene markers were highly skewed and well described by the fitted log-normal and Sichel functions (Fig. S2 A and B). The curve for the 16S rRNA gene was flatter than that for the *rpoB* gene, indicating a more skewed distribution. This effect was subtle, but it is reinforced by calculating the amount of sampling required to observe 90% of the true diversity [23] (Table 2). The choice of distribution has a large impact on required sampling effort, but for both distributions the median number of reads required is at least an order of magnitude larger for the 16S rRNA than for the *rpoB* gene. This result is significant in that the predictions for the two genes for the same choice of distribution lie outside of each other's confidence intervals (Table 2).

**Table 2:** 90% sampling effort, defined as number of reads required to observe 90% of the true diversity, for proteobacterial species.

marker (OTU cut-off)	90% sampling effortlog-normal distribution*	90% sampling effortSichel distribution*
*rpoB* (2.3%)	2.46e+07 : 7.15e+07 : 2.21e+08	4.27e+05 : 5.86e+05 : 8.65e+05
16S rRNA (1%)	5.36e+08 : 3.67e+09 : 1.63e+11	2.55e+06 : 4.16e+06 : 8.36e+06

* = The parametric total diversity estimates are given as lower 95% confidence interval : median : upper 95% confidence interval.

### Population Genetic Analyses

**Figure 3:**
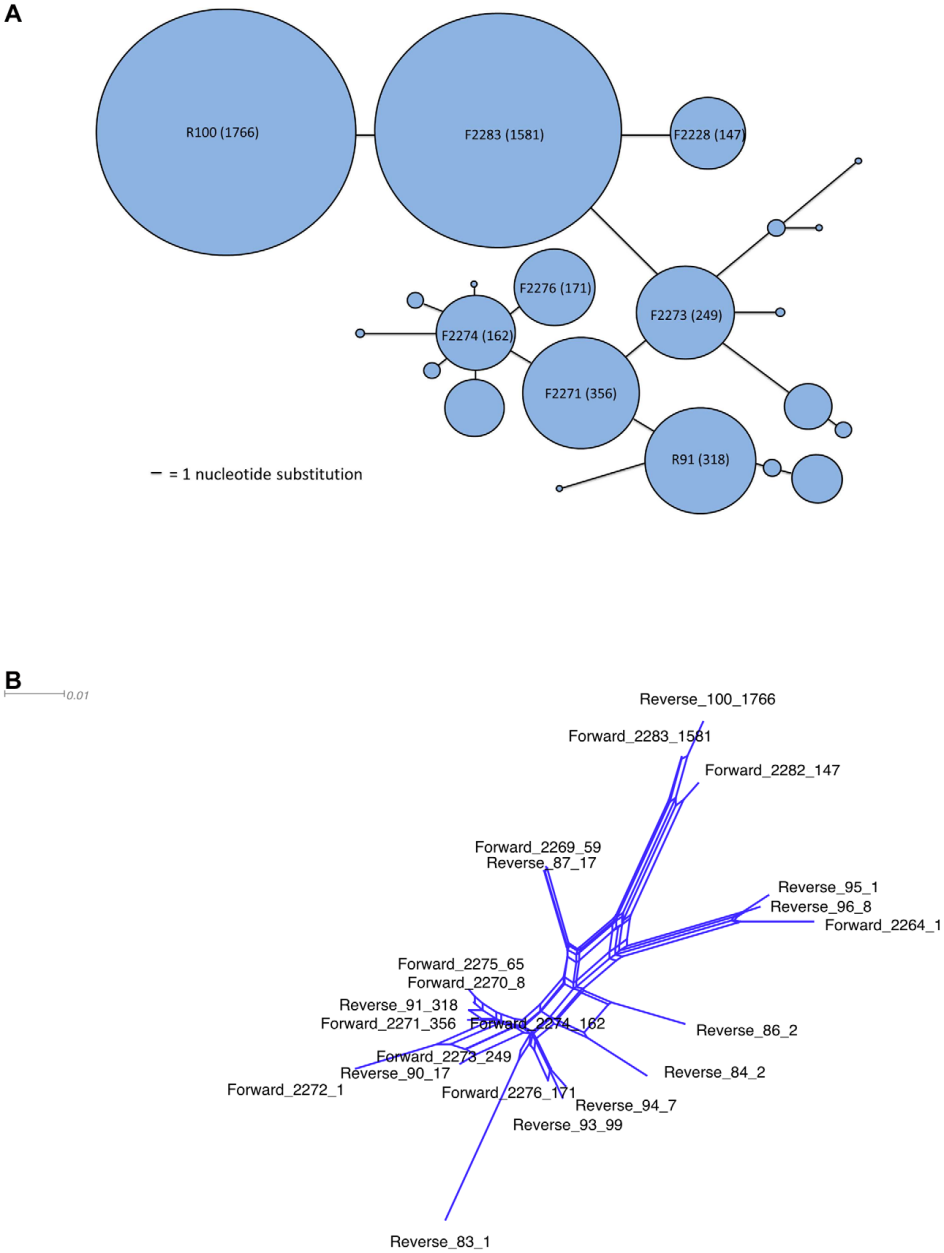
Population-level analyses. A: a Minimum Spanning Tree for all sequences more than 94% similar to abundant Anaeromyxobacter sequence R100. Circle size equates with the number of sequences, bar length equates with the number of nucleotide substitutions between sequences. B: a NeighbourNet phylogenetic network based on the same sequences as in A.

In order to demonstrate the suitability of pyrosequencing data from the given marker gene for analyses within a population-level framework, all sequences with more than 94% similarity to a particularly abundant sequence (1766 identical individuals, 82% nucleotide identity to both *Anaeromyxobacter dehalogenans* 2CP-1 and *Anaeromyxobacter* sp. K) were examined in more detail. First, a Minimum Spanning Tree was created (Fig. 3A). MST analysis is a useful tool for visualizing evolutionary relationships at the population level, where ancestral and evolved genotypes coexist (unlike phylogenetic trees, where extant genotypes are represented by the tips of branches and ancestors are represented by internal nodes). Such ‘population snapshots’ have been primarily generated with allelic MLST data using the eBurst algorithm [29], [30] (http://eburst.mlst.net/) but have also been applied to single-gene nucleotide data obtained from isolates (e.g. [31]).

The shape of the MST (Fig. 3A) does not point to an epidemic population structure, where a common, presumably well-adapted genotype is connected to multiple, less frequent ‘offshoot’ genotypes [32]. Instead, multiple common genotypes are all connected; such a ‘straggly’ appearance has been shown to be the result of high homologous recombination rates in eBurst-generated population snapshots [33]. It remains to be investigated whether recombination influences the MST algorithm used here in the same way, but a PHI test indicated highly significant levels of recombination in this dataset (p<0.0001). The influence of recombination is also indicated by the reticulated structure of the associated phylogenetic network (Fig. 3B).

Since proteobacterial diversity is so very high, most sequences in this dataset were separated by many substitutions. In order to truly zoom in on population-level processes, a much greater amount of sequencing would be needed and/or more specific primers would need to be employed. This will give better coverage below the species-level and allow for more options with respect to tests of recombination and selection. When well-characterized functional genes are chosen, it should also be possible to survey mutations of known effect on protein function. This was actually also possible for the fragment of *rpoB* sequenced here, as it covers a region (cluster II) where mutations conferring rifampicin resistance have been found in various species [34]. None of these mutations were found to be present in our dataset.

## Discussion

In this study, we sought to compare the merit of the protein-coding *rpoB* marker gene with that of the standard 16S rRNA marker gene for assessing bacterial community diversity by amplifying and pyrosequencing both genes from the same soil sample. Single-copy protein-coding genes essential for cell functioning have an ‘internal check’ in that any disruption of the reading frame found must be the result of experimental error. Roche 454 pyrosequencing has the advantage of yielding a large number of sequences, but it is also vulnerable to miscalls of homopolymer runs that cause frame shifts [15]. Denoising algorithms can mitigate this effect to a large extent, as this and previous studies [15], [17] have shown, but reading frame correction was found to provide a clear additional benefit (Fig. 1). Reading frame correction in *rpoB* was found to perform equally well as the combined use of denoising and reading frame correction for taxa above the species-level (Fig. 1). This correction step might prove especially useful for sequencing protein-coding genes using the Illumina method, for which no denoising program yet exists.

In any ecosystem survey, it is important to quantify diversity in such a way that it can be related to the basic unit of community diversity, the species, as unambiguously as possible. Current microbial ecology studies use 16S rRNA gene sequences as a proxy for species diversity. However, the divergence of *rpoB* correlates better to overall genomic divergence [7], allowing for the selection of a more reliable OTU cut-off. The observed richness (Table 1) and diversity (Fig. 2) of proteobacterial species was markedly higher when quantified using *rpoB*. This phenomenon is likely to be at least partly due to the effect of copy number, where individual bacteria add multiple identical (or highly similar) copies of 16S rRNA genes to the sequenced pool. Remarkably, however, the total estimated number of proteobacterial species did not differ between the two markers (Table 1). This implies that *rpoB* can be a more efficient marker than the 16S rRNA gene when a subset of total diversity is targeted, revealing more species for any population sample and requiring an order of magnitude fewer reads to obtain 90% of the true diversity (Table 2).

Although its lower level of conservation makes it less suited for high-level classification, *rpoB* offers greater resolution than the 16S rRNA gene, making it in theory a better marker to distinguish between strains and species. The greater resolution of protein-encoding housekeeping genes compared to ribosomal genes has been appreciated for some time, and many isolation-based studies exist employing the *rpoB* gene (using this gene either exclusively, e.g. [35], [36], [37], or using this gene in combination with other housekeeping genes in Multilocus Sequence Typing (MLST) studies, e.g. [38], [39], [40]). In addition, the *rpoB* gene and similarly conserved single-copy markers have been extracted from metagenomic datasets for specific analyses [41], [42].

Our study is one of the first to apply PCR-based pyrosequencing to a protein-coding gene from the environment (see also [43], [44]). Three main disadvantages are associated with the use of *rpoB* (or any other protein-coding marker). First, it is not conserved enough to be of use as a universal marker and only a subset of the microbial community can be targeted. Second, assigning taxonomy to the sequences is problematic because no appropriate databases and classifiers are available. Third, experiments using complex but defined communities are necessary to rigorously test primer bias (although this holds true for the 16S rRNA marker also). However, in comparison with the 16S marker, single-copy protein-coding genes offer several advantages: there is a possibility for additional error-correction, sampling efficiency could be higher, insights can be provided into population-level processes such as homologous recombination rate and mutations of known effect can be surveyed. Finally, with more advanced computing options becoming available, such data can be analyzed not only on the level of OTUs, but also directly at the sequence level, for instance in neutral models developed in population genetics [45].

## Supporting Information

Figure S1 **MEGAN classification of 16S RNA (red) and** ***rpoB*** **(blue) genes up to the Class level (normalized read frequencies).**
(TIF)

Figure S2 **The frequency of 16S rRNA 1% Proteobacteria OTUs (A) and** ***rpoB*** **2.3% Proteobacteria OTUs (B) with a given abundance.** The axes are log scaled and data points have been aggregated to reduce observed noise. We also show fits of the log-normal and Sichel distributions to this data.
(TIF)

Table S1 **Strains used to design the** ***rpoB*** **primers.**
(DOCX)
