# Supplementary figures and images for: A Comparison of rpoB and 16S rRNA as Markers in Pyrosequencing Studies of Bacterial Diversity
Source: PLoS One. 2012 Feb 15;7(2):e30600. doi: 10.1371/journal.pone.0030600 (PMC3280256; doi:10.1371/journal.pone.0030600)

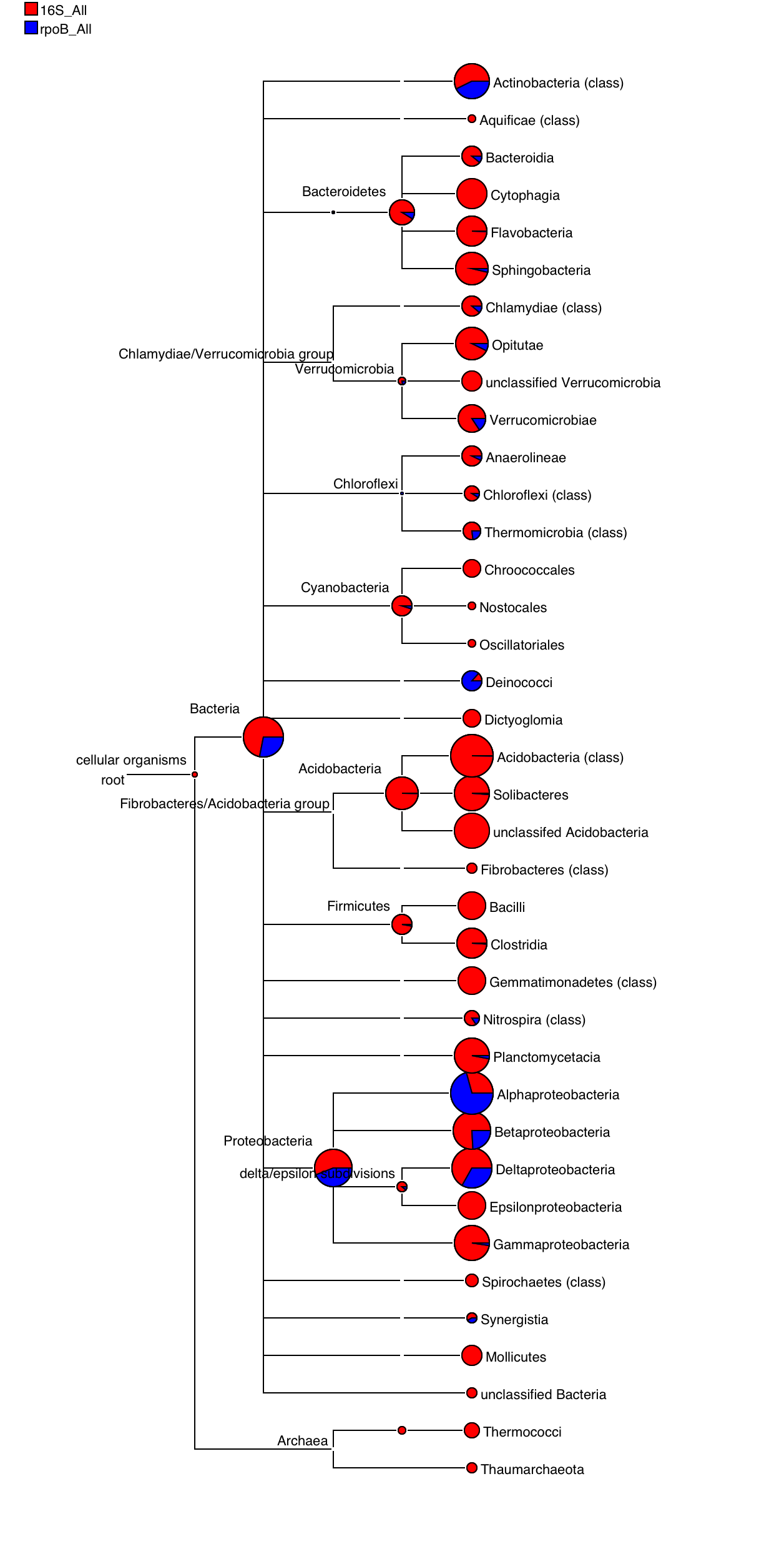

Supplement: Figure S1 — MEGAN classification of 16S RNA (red) and rpoB (blue) genes up to the Class level (normalized read frequencies). (TIF) [file pone.0030600.s001.tif]

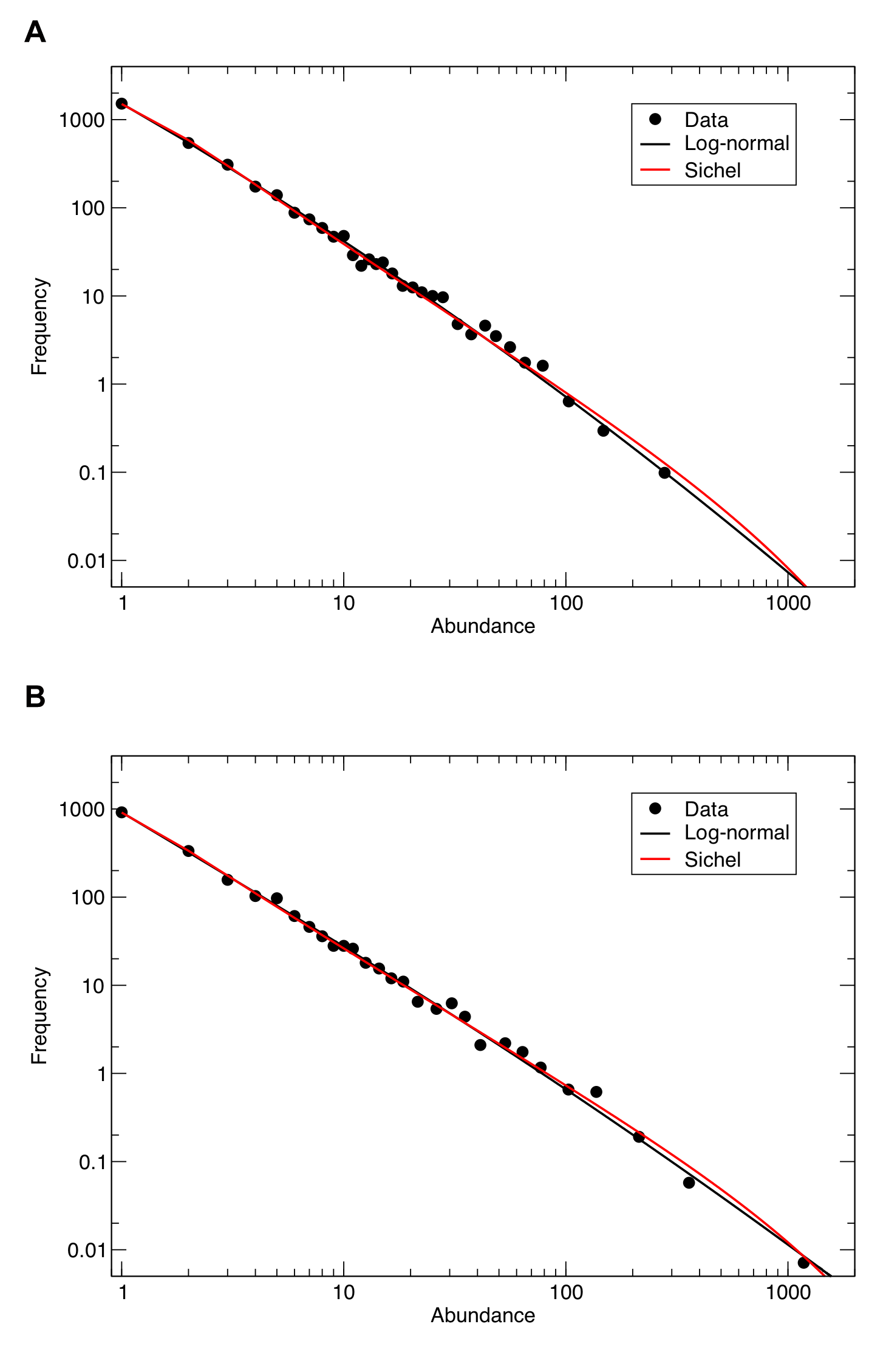

Supplement: Figure S2 — The frequency of 16S rRNA 1% Proteobacteria OTUs (A) and rpoB 2.3% Proteobacteria OTUs (B) with a given abundance. The axes are log scaled and data points have been aggregated to reduce observed noise. We also show fits of the log-normal and Sichel distributions to this data. (TIF) [file pone.0030600.s002.tif]
