# Supplementary material for: A Comparison of rpoB and 16S rRNA as Markers in Pyrosequencing Studies of Bacterial Diversity
Source: PLoS One. 2012 Feb 15;7(2):e30600. doi: 10.1371/journal.pone.0030600 (PMC3280256; doi:10.1371/journal.pone.0030600)
Supplement: Table S1 — Strains used to design the rpoB primers. (DOCX) [file pone.0030600.s003.docx]

Table S1. Strains used to design the *rpoB* primers

Class Genus Species Strain

Alfaproteobacteria *Ehrlichia chaffeensis* str. Arkansas

*Methylobacterium extorquens* AM1

*Anaplasma marginale* sSt.Maries

*Brucella melitensis* ATCC_23457

*Liberibacter asiaticus* psy62

*Oligotropha carboxidovorans* OM5

*Rhizobium etli*  CFN 42

*Rhodobacter sphaeroides*  2.4.1

*Rickettsia felis*  URRWXCal2

*Roseobacter denitrificans* OCh114

Betaproteobacteria *Azoarcus sp.*  EbN1

*Bordetella parapertussis*  12822

*Burkholderia mallei*  SAVP1

*Chromobacterium violaceum*  ATCC 1

*Herminiimonas arsenicoxydans* rpoB

*Neisseria meningitidis*  alpha14

*Ralstonia eutropha* H16

Deltaproteobacteria *Desulfobacterium autotrophicum*  HRM2

*Geobacter sulfurreducens*  PCA

*Myxococcus xanthus* DK1622

*Sorangium cellulosum* 56

Epsilonproteobacteria *Campylobacter jejuni*  RM1221

*Helicobacter hepaticus* ATCC5

*Nautilia profundicola* AmH

*Nitratiruptor* SB155-2

*Sulfurovum* NBC37-1

Gammaproteobacteria *Acinetobacter baumannii*  AB0057

*Aeromonas salmonicida* A449

*Aliivibrio salmonicida* LFI1238

*Azotobacter vinelandii* DJ

*Blochmannia floridanus*

*Buchnera aphidicola* APS

*Coxiella burnetii* CbuK Q154

*Francisella tularensis* FSC198

*Haemophilus influenzae* 86-028NP

*Legionella pneumophila*  Corby

*Pasteurella multocida* PM70

*Photorhabdus asymbiotica* ATCC43949

*Proteus mirabilis*  HI4320
